# Midfrontal theta dynamics index the monitoring of postural stability

**DOI:** 10.1093/cercor/bhac283

**Published:** 2022-09-06

**Authors:** Mitchel Stokkermans, Teodoro Solis-Escalante, Michael X Cohen, Vivian Weerdesteyn

**Affiliations:** Radboud Universitary Medical centre for Medical Neuroscience, Department of Rehabilitation, Reinier Postlaan 4, 6525 GC Nijmegen, The Netherlands; Donders Institute for Brain cognition and behavior, Department of synchronisation in neural systems Kappitelweg 29,6525 EN Nijmegen, The Netherlands; Radboud Universitary Medical centre for Medical Neuroscience, Department of Rehabilitation, Reinier Postlaan 4, 6525 GC Nijmegen, The Netherlands; Donders Institute for Brain cognition and behavior, Department of synchronisation in neural systems Kappitelweg 29,6525 EN Nijmegen, The Netherlands; Radboud Universitary Medical centre for Medical Neuroscience, Department of Rehabilitation, Reinier Postlaan 4, 6525 GC Nijmegen, The Netherlands; Sint-Maartenskliniek Research, Hengstdal 3, Ubbergen, 6574 NA Nijmegen, The Netherlands

**Keywords:** balance monitoring, action monitoring, EEG, midfrontal theta

## Abstract

Stepping is a common strategy to recover postural stability and maintain upright balance. Postural perturbations have been linked to neuroelectrical markers such as the N1 potential and theta frequency dynamics. Here, we investigated the role of cortical midfrontal theta dynamics of balance monitoring, driven by balance perturbations at different initial standing postures. We recorded electroencephalography, electromyography, and motion tracking of human participants while they stood on a platform that delivered a range of forward and backward whole-body balance perturbations. The participants’ postural threat was manipulated prior to the balance perturbation by instructing them to lean forward or backward while keeping their feet-in-place in response to the perturbation. We hypothesized that midfrontal theta dynamics index the engagement of a behavioral monitoring system and, therefore, that perturbation-induced theta power would be modulated by the initial leaning posture and perturbation intensity. Targeted spatial filtering in combination with mixed-effects modeling confirmed our hypothesis and revealed distinct modulations of theta power according to postural threat. Our results provide novel evidence that midfrontal theta dynamics subserve action monitoring of human postural balance. Understanding of cortical mechanisms of balance control is crucial for studying balance impairments related to aging and neurological conditions (e.g. stroke).

## Introduction

Maintaining balance is crucial for humans, and indeed, falling due to reduced balance ability is a serious health risk for the elderly and individuals with movement disorders such as Parkinson’s disease. Postural stability is challenged with every intentional movement and by unpredicted changes in our surroundings. The central nervous system has a remarkable ability to monitor balance and sudden postural changes and rapidly engage corrective postural responses (Jacobs and Horak 2007; Maki and McIlroy 2007; Bolton 2015).

The traditional view was that balance is mainly regulated by subcortical brain regions (Sherrington 1910; Diener et al. 1984). However, it has become increasingly clear that the cerebral cortex also contributes to postural control (Jacobs and Horak 2007; Nutt et al. 2011; Jacobs 2014; Takakusaki 2017). The cortex may contribute by monitoring deviations from a desired stable posture, related to an internal model of postural stability (Forbes et al. 2018), and by initiating corrections, for example through regulation of subcortical excitability of the automatic postural response or through direct corrective responses at later-phase response latency (Bolton 2015).

Balance studies with electroencephalography (EEG) recordings identified 2 key electrophysiological signatures of cortical involvement in balance control. The N1 potential is a robust negative event-related potential occurring at ~150 ms following a balance perturbation and is presumably related to sensory integration and cognitive processes (Dietz et al. 1985; Goel et al. 2018; Payne et al. 2019). Time–frequency analysis shows a strong increase over multiple frequency bands, of which the theta frequency band (3–8 Hz) dominates, suggesting a strong relationship between the N1 potential and the underlying theta dynamics (Sipp et al. 2013; Marlin et al. 2014; Varghese et al. 2014; Hülsdünker et al. 2015). The event-related nature and scaling of N1/theta with perturbation intensity suggest involvement in sensory processing (Jacobs and Horak 2007; Maki and McIlroy 2007; Bolton 2015). However, these indices are not simply sensory markers: the N1 is modulated by anticipated perturbations (Adkin et al. 2006), and both N1 and theta modulate according to ensuing recovery behavior, with theta power better describing the variance within the data compared to the N1 amplitude (Solis-Escalante et al. 2020). The latter is of particular interest, as it hints at midfrontal theta dynamics playing a role in balance monitoring and predicting the need for a corrective step. Yet, the intimate relationship between both strong perturbation accelerations and stepping behavior obscures the interpretation of theta involvement in balance monitoring, because stronger perturbations are more likely to elicit step responses which does not directly imply the monitoring of balance.

While monitoring balance and predicting the need for corrective responses, the CNS may use an internal representation of the postural changes and body dynamics. The predictive coding theorem is proposed to be involved in the monitoring of upright stance (Yao and Levine 2009), by comparing external sensory postural information with expected sensory information stored in an internal model of stability (Forbes et al. 2018; Rasman et al. 2018). A mismatch between these 2 results in a prediction error of the sensory information stored in the internal model. It has been suggested that the transient increase of theta power is representative of the prediction error and, therefore, indicates the deviation of current and expected posture.

Here, we manipulated the relationship between acceleration and stepping behavior by altering the initial standing posture, which changes the distance between the center of mass (CoM) and the boundaries of the base of support prior to perturbations. Therefore, perturbations with identical intensities would differently impact postural stability depending on the direction of perturbation and the initial posture. If midfrontal theta indeed subserves an action monitoring role of postural balance, different initial leaning postures should modulate midfrontal theta dynamics. In the event of a balance perturbation, the internal processes of action monitoring must identify the impact of the perturbation in accordance with the internal model of postural stability and initiate an appropriate corrective response. When manipulating the initial postural state, the internal model of stability may be updated to correctly represent the current postural state and evaluate the threat imposed by a given perturbation. The evaluation of the perturbation’s impact on stability may then be reflected in the midfrontal theta dynamics.

Our goal was to investigate the participation of midfrontal theta dynamics in a behavioral monitoring system for reactive balance responses. Based on previous findings implicating midfrontal theta in action monitoring, we hypothesized that midfrontal theta reflects the phasic activation of a cortical system that monitors balance and signals threats to postural stability (i.e. we expect that theta dynamics are stronger when the leaning direction and perturbation direction are congruent). To test this hypothesis, we recorded EEG and motion tracking data while participants were instructed to try to maintain their balance with feet-in-place responses following sudden movements of the support surface. Furthermore, we instructed participants to lean forward or backward to manipulate their initial stability state and, thereby, manipulate the impact of a given perturbation on postural stability.

## Materials and methods

### Participants

Twenty young healthy adults (10 female; age mean 23.9 years, SD 3.6 years) participated in this study. All participants received ample information about the experiment. In addition, all participants were naïve participants and did not have previous experience on the platform. Afterwards, the participants voluntarily signed an informed consent form and were financially compensated after completion of the study. None of the participants had previous history of neuromuscular disease or any other impairment that could affect their performance in the experiment. The experimental procedure was approved by the Research Ethics Committee of the Radboud University Medical Center (Nijmegen, The Netherlands; Dossier 2018-4970). The experiments were conducted in line with the Declaration of Helsinki.

### Experimental paradigm

**Fig. 1:**
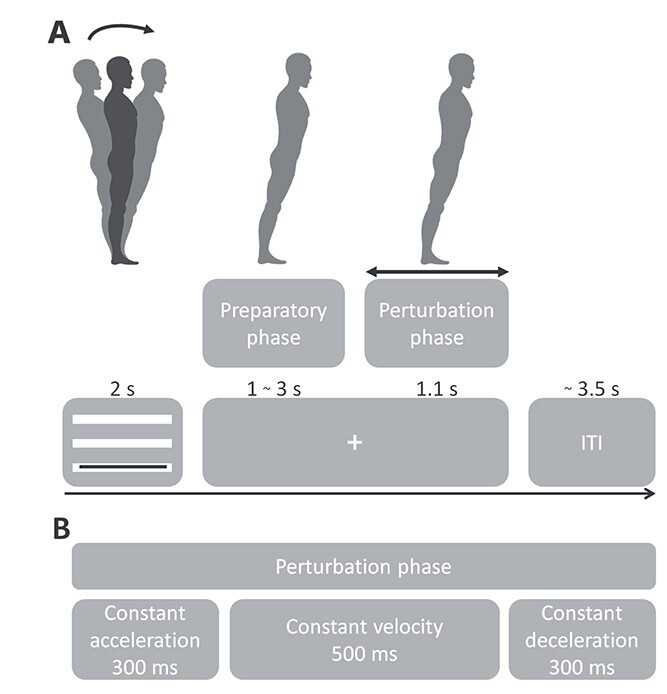
Experimental procedure. A) Participants were instructed to maintain 1 out of 3 postures during a sequence of perturbations. Perturbations were randomized in onset time, direction, and acceleration. The initial posture prior to perturbation was fixed per sequence. A trial consisted of a visual cue indicating the continuous leaning angle for 2 s, followed by a fixation cross. Platform onset was randomized from 1 to 3 s and the perturbation lasted for 1.1 s. After a perturbation, the platform slowly returned to the initial position in ~3.5 s. At platform return, the visual feedback of initial leaning angle was presented. Visual feedback for leaning posture was presented through 3 white bars representing forward leaning (bottom bar), neutral stance (middle bar), and backward leaning (top bar). A black bar presented in front of the white bars represented the participant’s real-time leaning angle. Participants were instructed to keep the black bar on the white bar corresponding to the instructed leaning direction. The fixation cross was presented at the same height as the leaning bar for that sequence to guide the participant. Initial leaning posture had to be maintained throughout the fixation cross period, ensuring that postural stability was controlled at platform perturbation. B) Platform perturbation profiles.

Participants were familiarized with the experimental procedure through a series of 28 forward and backward perturbations with increasing acceleration, as delivered by the Radboud Falls Simulator (Nonnekes et al. 2013a; Solis-Escalante et al. 2019, 2020). Participants stood barefoot on the movable platform with their feet at shoulder width and the arms crossed in front of the body. They were instructed to do their best to keep the feet-in-place in response to a balance perturbation. Platform perturbation profiles consisted of 300 ms platform acceleration, 500 ms constant velocity, and 300 ms deceleration (Fig. 1B). Platform accelerations were randomized and ranged from 0.25 to 1.9 m/s^2^ with a higher resolution at lower accelerations in both forward and backward perturbation directions (0.25, 0.4, 0.7, 1.0, 1.3, 1.6, 1.9 m/s^2^). This distribution was chosen, because we expected theta modulations to be more pronounced during the feet-in-place responses at lower platform accelerations, as illustrated in our previous study (Solis-Escalante et al. 2020). In addition, this distribution allowed us to also record a fair number of feet-in-place responses in the conditions with congruent leaning and perturbation directions (i.e. those with a greater necessity for stepping responses). Forward translation of the platform elicited postural sway and an eventual step in the backward direction; similarly, backward translation of the platform elicited postural sway and an eventual step in the forward direction. Henceforth, we refer to the movement of the body with respect to the feet, meaning that in the forward perturbation direction, the body moved in the forward direction and backward perturbations moved the body in the backward direction. Prior to a sequence, participants were instructed to maintain a leaning posture throughout the whole sequence. In total, participants underwent 5 sequences of each 3 leaning conditions. Leaning conditions were altered from neutral stance to forward leaning and then backward leaning followed again by neutral stance to prevent fatigue due to the leaning posture. The experiment consisted of 15 sequences containing 29 balance perturbations each (435 total). The first perturbation of a sequence always consisted of a low-intensity dummy trial and was not included in the analyses, resulting in 420 trials for analysis per participant.

To control the initial posture, participants received feedback with respect to their leaning angle, which was computed as the angle between reflective markers located at the left and right ankle and the seventh cervical vertebra. Neutral stance was assigned to a 90° angle, whereas forward leaning was set to 85° and backward leaning to 95° of lean. Participants were instructed to maintain a straight posture, preventing any flexion at the knee or hip while maintaining the leaning position. Prior to each perturbation, participants received real-time visual feedback of their leaning angle, which was presented on a projection screen in front of the participants for a fixed 2 s (Fig. 1A). The feedback was replaced with a fixation cross 1–3 s prior to the perturbation onset and remained on the screen throughout the trial. Real-time data stream allowed the experimenter to control participants’ posture and performance, such as maintaining leaning angle, excessive knee flexion, and changes in leg weight bearing (which may indicate the use of specific strategies to counteract balance perturbations).

To prevent fatigue, participants took a small break of 5 min after 3 consecutive sequences and were seated on a chair. After 9 sequences, participants were given a 20-min lunch break. The active experiment time was 2.5 h and the preparation time was 2.5 h, the complete lab visit lasted a maximum of 6 h (including resting breaks).

### Data acquisition

We recorded high-density EEG using a cap with 126 Ag-AgCl electrodes (WaveGuard, ANT Neuro, The Netherlands). The electrodes were fixed in the cap and distributed across the scalp according to the 5% electrode system (Oostenveld and Praamstra 2001). The EEG was referenced to the common average during acquisition. The ground electrode was placed on the left mastoid. A biosignal amplifier (REFA System, TMSi, The Netherlands) recorded the EEG at 2,048 Hz without any filters, except for a hardware low-pass filter at 552 Hz. To monitor physiological activity that could present artifacts in the EEG, we also recorded electrical activity of the left eye in the vertical and horizontal directions (electrooculogram, EOG) using adhesive Ag-AgCl electrodes. The EOG was recorded from electrodes placed slightly under the left eye (vertical eye movement) and at the outer canthus of the left eye (horizontal eye movement).

Body movements were recorded using an 8-camera 3D motion analysis system (Vicon Motion Systems, United Kingdom) at a sample rate of 100 Hz. For this purpose, a total of 23 reflective markers (PlugInGait Full-body AI model excluding the head and arm markers; Vicon Nexus software 2.7.1) were attached to anatomical landmarks on the participants’ body.

Ground reaction forces were recorded from 2 force plates (AMTI Custom 6 axis composite force platform, USA; size: 60 × 180 cm each; sampling rate: 2,000 Hz) embedded in the moveable platform. Trials were recorded from −2 to +5 s relative to the platform perturbation. Synchronization triggers were generated by the platform controller and recorded for post hoc alignment of motion data via Vicon, and EEG signals via the EEG acquisition computer.

### Kinematic and behavioral data processing

Force plate data were used to identify the moment of foot-off (step onset) during a trial. The threshold for foot-off identification was set at a participant-specific level of 80% unloading of the weight borne on one leg. Steps occurring before 1.1 s post perturbation onset (i.e. termination of platform movement) were included in the analysis as stepping trials. A total of 194 trials (2.4% of total) with steps occurring outside this temporal window were excluded from further analysis.

Kinematic data were preprocessed using Vicon Nexus software (Vicon Motion Systems, UK, version 2.7.1). Markers were labeled and reconstructed in a batch preprocessing pipeline. Occluded markers on the hip or trunk segment were gap filled with a rigid body fill algorithm when at least 3 other markers of the body segment were visible. Other missing markers were gap filled using the Vicon Woltring filter, but only if they did not overlap platform perturbation onset (±0.5 s) and lasted a maximum of 50 frames. Marker data were imported to MATLAB and 10 Hz low-pass filtered (fifth order Butterworth IIR filters, zero-phase shift). Relative marker movement was computed by subtracting the platform marker movement from each individual body marker and imported to the EEG dataset.

#### Data analysis: stepping probability

The stepping probability quantifies the distribution of feet-in-place and stepping responses across platform acceleration. The responses were coded as feet-in-place (0) and stepping (1) responses and the probability of a stepping response is expressed in the ratio of step responses and the total number trials recorded at that specific acceleration and leaning condition. A stepping probability of zero indicated that stepping responses did not occur, whereas a probability of 1 indicated that steps occurred in all trials.

#### Data analysis: margin of stability

The margin of stability (MoS) (Hof et al. 2005; McAndrew Young et al. 2012) is a kinematic measure that reflects dynamic stability, by taking into account both the position and the velocity of the horizontal CoM projection relative to the boundary of the support base (BoS). We obtained the CoM from the Vicon processed data and the BoS was determined by averaging the anterior posterior position of the toes in the forward perturbation direction and the heels in the backward perturbation direction. A low MoS indicates a less stable postural state as the CoM may be approaching the BoS. A MoS value of zero means there is no more room for the CoM to move before extending beyond the BoS and a negative value of MoS indicates that the CoM has traveled beyond the BoS. We calculated the MoS at 300 ms post perturbation onset, where the platform acceleration transitioned in a constant velocity. We used a fixed single-trial value for the BoS averaged over −2 to −1 s prior to perturbation, since we were only interested in dynamics prior to foot off. The MoS allowed us to verify whether the leaning conditions in combination with platform accelerations indeed posed different postural threats. The MoS was calculated according to Hof et al. (2005).

### E‌EG processing

EEG and EOG data were preprocessed in MATLAB using functions of the EEGLAB toolbox (Delorme and Makeig 2004). Data were bandpass filtered (2–200 Hz, consecutive high-pass and low-pass fifth-order Butterworth IIR filters, zero-phase shift) using the filtfilt.m matlab function and averaged referenced. Continuous data were cut into epochs of 5 s (−2 to +3 s relative to perturbation onset) and concatenated per participant for further processing. Channels were flagged for rejection based on a kurtosis >3 and a variance >3 and rejected based on visual inspection (mean 5.5, SD 6.3 rejected channels). Additionally, epochs were rejected through visual inspection for noise. An independent component analysis (Infomax ICA) with a minimum of 90 and maximum of 126 principal components (depending on the rank of the EEG data) was run and independent components were rejected based on being excessively noisy and of non-brain origin (mean 91 rejected components, SD 16 rejected components). Artifact-reduced EEG was obtained by back projection of the retained independent components.

#### Generalized eigendecomposition

We applied a generalized eigendecomposition (GED), a multivariate source-separation method, on the clean EEG data in order to derive a spatial filter that is optimized for theta (3–8 Hz) activity (Cohen 2017). Computation of the GED on fewer components resulting from ICA cleaning does not reduce the rank of the GED as GED is defined for any rank matrix (Cohen 2021). Two covariance matrices are constructed corresponding to theta-filtered data (matrix S) and broadband data (matrix R) (Equation 1). The GED on these 2 matrices returns a set of eigenvectors (in matrix W) and corresponding eigenvalues (in matrix $\varLambda$), and the eigenvector associated with the largest eigenvalue is a set of channel weights that maximizes the relative energy in the theta band.


(1) \begin{equation*} \mathrm{SW}=\mathrm{RW}\Lambda \end{equation*}

This generalized eigenvalue equation solves the Rayleigh quotient and is often used in machine learning and brain–computer interface research (Blankertz et al. 2008; Haufe et al. 2014; Cohen 2021).

The eigenvector with the (i) greatest eigenvalue, (ii) ERP average, and (iii) midline/midfrontal scalp topography was selected per participant for further analysis of the EEG data. The component time series was computed as w^T^X, where *X* is the channel time series data, and the spatial map was computed as w^T^S (Haufe et al. 2014).

#### Time–frequency analysis

The neural time series data after GED analysis were further characterized through time–frequency decomposition. This was implemented by narrow-band filtering the time series at a range of frequencies through trial-by-trial convolution with complex Morlet wavelets. Equations (2) and (3) show the construction of the complex Morlet wavelets.


(2) \begin{equation*} {\varPsi}_f={e}^{i2\pi ft}{e}^{-{t}^2/2{s}^2} \end{equation*}

(3) \begin{equation*} s=\frac{n}{2\pi f} \end{equation*}

Where $t$ represents time, $f$ is frequency, $s$ is width of the Gaussian modulating the complex sine wave, and $n$ stands for the number of wavelet cycles. The number of cycles per wavelet controls the temporal and spectral precision tradeoff. We used 40 frequencies linearly spaced between 2 and 60 Hz. Wavelet widths were logarithmically spaced from 4 to 12 cycles.

For the visualization of average time–frequency data, we baseline corrected the data with a baseline window of 1 to −0.2 s prior to perturbation onset.

### Single-trial EEG theta power calculations

Preprocessed single-trial EEG time series data were filtered in the 3–8 Hz range, (consecutive high-pass and low-pass fifth-order Butterworth IIR filters, zero-phase shift) and theta power was computed as the magnitude of the Hilbert transform. Applying the Hilbert transform to the data was done over the whole trial timeseries of 4 s, making sure that the timeseries was large enough to compare theta power over a whole wave cycle. Temporal single trial averaging over 0.1–0.3 s post perturbation was done after using the Hilbert transform in the theta band filtered data. This means that when averaging over a smaller time window, this accurately represents the theta power at that time, even though the theta oscillations may be related to a longer lasting event. Trials with step responses occurring in the 0.1–0.3 s epoch were rejected to reduce potential step-related EEG interference. We did not subtract single-trial baseline power due to single-trial instability. Instead, single-trial theta power was normalized by computing the *z*-score over all trials per participant before statistical analysis. To verify that only theta dynamics scale with balance monitoring behavior, we similarly treated and analyzed the data in the alpha (9–12 Hz) and beta (15–25 Hz) frequency range.

### Statistical analysis

#### Data quality check (EEG)

To determine whether EEG data quality was comparable for all leaning conditions, we computed single-trial variance and determined any effects of leaning conditions using a one-way ANOVA.

#### Leaning condition-specific average theta baseline power

Time–frequency average theta baseline dynamics were tested for an effect of leaning with a one-way ANOVA to indicate whether leaning conditions had an effect on theta dynamics prior to perturbation.

#### Theta dynamics (EEG)

EEG data were analyzed separately for forward and backward directions with 2 generalized linear mixed-effects models (GLME) with factors Acceleration, Stepping behavior, and Leaning condition. For the categorical factor Stepping behavior, the feet-in-place responses were dummy coded as zero, whereas the categorical factor Leaning condition was dummy coded per perturbation direction according to the most stable condition, i.e. the condition associated with a greater proportion of feet-in-place responses. Therefore, backward leaning feet-in-place responses were considered the most stable condition in the forward perturbation direction model and forward leaning feet-in-place responses in the backward perturbation direction model.

The GLME models are described by:


(4) \begin{eqnarray*}&& {Y}_{ij}={\beta}_0+{\mu}_{0j}+\left({\beta}_1+{\mu}_{1j}\right){A}_{ij}+\left({\beta}_2+{\mu}_{2j}\right){S}_{ij}\\&&\nonumber+\left({\beta}_3+{\mu}_{3j}\right){L}_{ij}+{\beta}_4{A}_{ij}{L}_{ij}+{\beta}_5{A}_{ij}{S}_{ij}+{\beta}_6{S}_{ij}{L}_{ij}\\&&\nonumber+{\beta}_7{A}_{ij}{L}_{ij}{S}_{ij}+{\varepsilon}_{ij} \end{eqnarray*}

Here, ${\beta}_0$ is the fixed intercept, ${\mu}_{0j}$ the random intercept, ${\varepsilon}_{ij}$ is the error term, ${\beta}_1$ is the fixed effect slope for acceleration ${A}_{ij}$, and ${\mu}_{1j}$ is the random slope for acceleration ${A}_{ij}$, with ${\mu}_{1j}$ assumed to be normally distributed with mean 0 and variance ${\sigma}_{\mu 1}^2$. ${\beta}_2$ is the fixed effect slope for stepping ${S}_{ij}$, and ${\mu}_{2j}$ is the random slope for stepping ${S}_{ij}$, with ${\mu}_{2j}$ assumed to be normally distributed with mean 0 and variance ${\sigma}_{\mu 2}^2$. ${\beta}_3$ is the fixed effect slope for leaning ${L}_{ij}$, and ${\mu}_{3j}$ is the random slope for leaning ${L}_{ij}$, with ${\mu}_{3j}$ assumed to be normally distributed with mean 0 and variance ${\sigma}_{\mu 3}^2$. ${\beta}_4$ is the fixed interaction effect slope for acceleration ${A}_{ij}$ and leaning ${L}_{ij}$, ${\beta}_5$ is the fixed interaction effect slope for acceleration ${A}_{ij}$ and stepping ${S}_{ij}$, and ${\beta}_6$ is the fixed interaction effect slope for stepping ${S}_{ij}$ and leaning ${L}_{ij}$. ${\beta}_7$ is the fixed 3-way interaction effect slope for acceleration ${A}_{ij}$, leaning ${L}_{ij}$, and stepping ${S}_{ij}$. The null hypothesis can be rejected if the confidence interval of a given regression coefficient does not include zero. The regression analyses were conducted at group level using single trials from all participants, after participant-specific normalization (z-score across trials) of the cortical theta power.

#### Behavioral parameters

To test whether stepping probability was changed between leaning angles, the stepping probability was analyzed with a nested logistic regression of individual stepping probability values with platform “Acceleration” and “Leaning condition” as the independent variables. The MoS was analyzed with a GLME with factors “Acceleration” and “Leaning condition.” See Equation 5 (as a simplified equation without the factor of Stepping from Equation 4).


(5) \begin{equation*} {Y}_{ij}={\beta}_0+{\mu}_{0j}+\left({\beta}_1+{\mu}_{1j}\right){A}_{ij}+\left({\beta}_2+{\mu}_{2j}\right){L}_{ij}+{\beta}_3{A}_{ij}{L}_{ij}+{\varepsilon}_{ij} \end{equation*}

These statistical analyses were conducted to corroborate the effect of the experimental manipulation (changes to initial postural stability due to leaning posture) on the distribution of reactive stepping behavior across the experimental conditions.

## Results

### Behavioral data

**Fig. 2:**
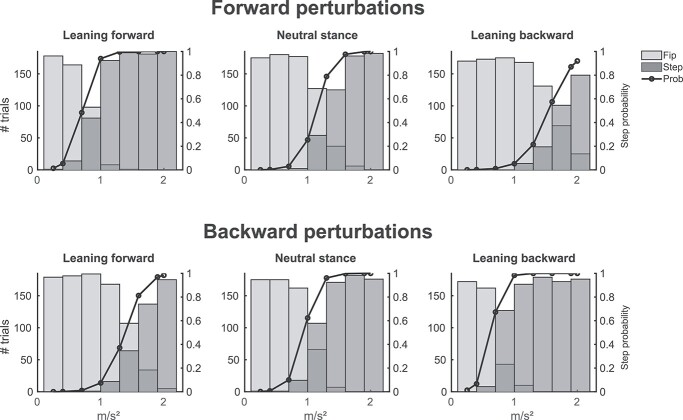
Group-level stepping probability. The top row histograms illustrate the forward-directed perturbation trial distribution for feet-in-place (Fip, light gray) and stepping (Step, dark gray) responses over the conditions forward leaning (left), neutral stance (middle), and backward leaning (right). The bottom row shows the trial distribution for backward-directed perturbation trials. The black curves represent the stepping probability per condition. Increased platform acceleration led to an increased stepping probability. Additionally, stepping probability increased when the leaning direction was congruent to the perturbation direction.

A total of 7,424 trials over 20 participants were analyzed from a total of 7,972 recorded trials. Five trials were rejected based on stepping prior to perturbation onset. A total of 270 trials were rejected based on steps occurring after the constant velocity phase of the motion platform. We rejected 195 trials based on artifacts in kinematic data and an additional 78 trials for artifacts in EEG data (see Methods section for trial rejection criteria). The forward perturbation direction analyses contained 3,715 trials, of which 2,062 were feet-in-place and 1,653 were step responses. The backward perturbation direction model contained a total of 3,709 trials with 1,830 feet-in-place and 1,879 step responses. Overall, the proportion of stepping responses for forward and backward responses were significantly different (${\chi}^2$ = 28.29, df = 3, *P* = 1.05e^−7^; forward 43% and backward 51%) with more steps being taken in the backward direction, see Fig. 2 for the detailed distribution over the different accelerations and leaning conditions. Step onsets were earlier in the backward perturbation direction (median = 376 ms, SD = 0.20 ms) compared to the forward perturbation direction (median = 440 ms, SD = 0.18 ms) (Mann–Whitney *U* test, *Z* = 14.91, *P* = 2.71e^−50^).

### Stepping probability

Figure 2 shows the stepping probabilities for both perturbation directions and the different leaning conditions. The results indicate that stepping probability was affected by both leaning manipulation and platform acceleration, meaning that increased platform accelerations led to an increased stepping probability and when leaning condition and perturbation direction were congruent, stepping probabilities were closer to one. Stepping probability distributions of forward and backward perturbation directions at neutral stance are similar to previous studies, indicating that the random selected group of participants did not under- or overperform the experiment (de Kam et al. 2016; Solis-Escalante et al. 2019).

The logistic regression model of the stepping probability in the forward perturbation direction showed an interaction of acceleration and congruent leaning condition (*t* = −4.01, dof = 3711, *P* = 6.04e^−5^). This indicates that stepping probability increases with greater platform accelerations and that forward leaning additionally shifts the greater stepping probability to lower platform perturbation accelerations. Differences in stepping probability due to the imposed leaning conditions are mainly observed over the middle range of accelerations with 50% stepping probability varying from ~0.7 m/s^2^ in the forward leaning condition to ~1.6 m/s^2^ in the backward leaning condition. The backward perturbation direction logistic regression model showed an interaction effect for “Leaning condition” and “Acceleration” (*t* = −6.21, dof =3705, *P* = 5.27e^−10^) with a main effect for both “Leaning condition” (*t* = 0.30, dof = 3705, *P* = 4.88e^−6^) and “Acceleration” (*t* = −8.99, dof = 3705, *P* = 2.55e^−19^). Once again, the differences in stepping probability due to the imposed leaning conditions are mainly observed over the middle range of accelerations with 50% stepping probability varying from ~1.3 m/s^2^ in the forward leaning condition to ~0.7 m/s^2^ in the backward leaning condition.

### Margin of stability

**Fig. 3:**
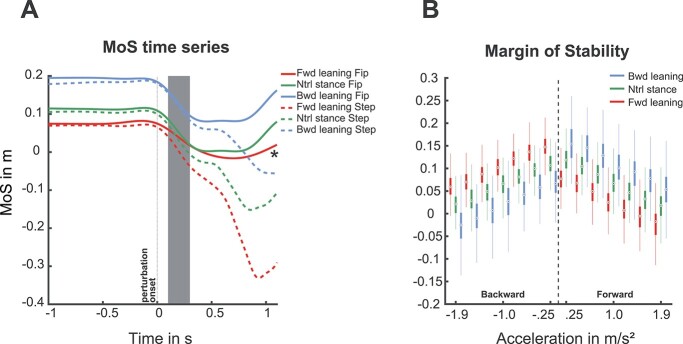
MoS. A) Single-trial MoS timeseries data of forward perturbations at 1 m/s^2^ (arbitrarily chosen from the experiment of one participant). Note that the MoS trajectory for the forward leaning feet-in-place trial (red solid line, indicated with an asterisk) was collected at an intensity of 0.7 m/s^2^, because no participant responded feet-in-place to 1 m/s^2^. Gray shaded area indicates time window of interest for theta analysis (100–300 ms). MoS values for further analysis were obtained from 300 ms post perturbation. B) MoS at 300 ms post perturbation for each leaning condition per acceleration. MoS illustrated over leaning conditions; forward leaning (red), neutral stance (green), and backward leaning (blue). The boxplots presented to the left of the dotted line represent MoS for backward perturbation directions, and the boxplots to the right of the dotted line represent the forward perturbation directions. The figure clearly illustrates that the leaning manipulation shifted the MoS for each individual acceleration (observed as the 3 boxplot colors per acceleration bin). Additionally, the MoS is reduced with increasing acceleration, which indicates a greater risk of losing postural stability.

In Fig. 3A, single-trial MoS timeseries are presented for all leaning conditions with a feet-in-place and step response in the forward perturbation direction at 1 m/s^2^ (^*^ = The forward leaning feet-in-place response trial intensity was 0.7 m/s^2^, solid red line). Our results indicate that overall step trials reach lower and, in some cases, negative values of MoS compared to feet-in-place trials. Step latencies did not occur during the initial 300 ms post perturbation; therefore, we analyzed MoS dynamics at 300 ms to determine leaning-induced differences on MoS.

Figure 3B shows the MoS for both perturbation directions and the different leaning conditions. The results indicate that the MoS is influenced by the acceleration and leaning conditions, in both the forward and backward perturbation directions. In addition, within acceleration bin of a leaning condition, MoS was overall greater in the forward compared to backward perturbation direction. In accordance with the stepping probability, when leaning condition and perturbation direction were congruent, the MoS was smaller (and stepping probability was closer to one). This is also indicated by the interaction effects of leaning condition and acceleration of the regression models. The forward perturbation direction model (*R*^2^ = 0.88; *F*(3439) =2,529; *P* = 8.94e^−204^) showed an interaction effect for the “Forward leaning condition × Acceleration” (${\beta}_3$ = −3.89, CI: [−19.73: 11.94], *P* = 0.016) and a main effect for the “Neutral leaning condition” (${\beta}_2$ = −36.83, CI: [−44.08: 29.57], *P* = 4.97e^−23^). The backward perturbation direction model (*R*^2^ = 0.90, *F*(3434) = 2,782, *P* = 1.80e^−284^) showed an interaction effect for both “Neutral leaning condition × Acceleration” (${\beta}_3$ = −17.7, CI: [−31.52: 3.89], *P* = 0.01) and “Backward leaning condition × Acceleration” (${\beta}_3$ = −36.35, CI: [−55.04: −17.67], *P* = 1.3e^−4^).

### Electroencephalography

Overall, EEG data quality was unaffected by leaning conditions as we found no effect on condition-specific trial variance (*F*(2) = 1.05, *P* = 0.35). Only 2 participants showed extensive noise, resulting in a greater amount of rejected components after ICA (mean 91 rejected components, SD 16 rejected components). Overall, ICA infomax was an appropriate method for over 95% of the participants and therefore considered suitable for the analysis pipeline. All leaning steps were conducted prior to data analysis to prevent any bias toward the results.

### GED topographies and time–frequency characteristics

**Fig. 4:**
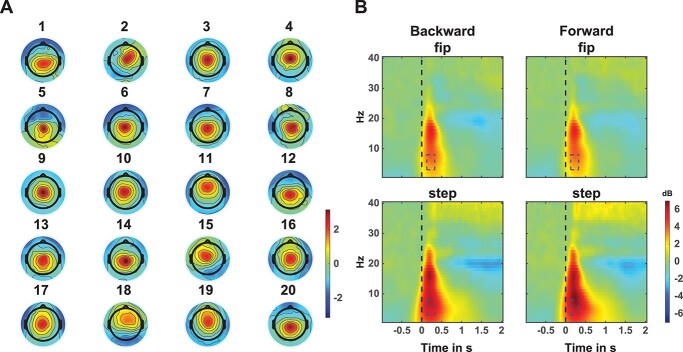
EEG data. A) Participant-specific scalp topographies of the eigenvector with the largest eigenvalue and a midfrontal topography. The scalp topography data were normalized per participant. B) Average time–frequency plots of 20 participants scaled power in dB. Plots are averaged over all leaning conditions. Top row panels show feet-in-place responses (fip), while bottom row panels show step responses. The left column illustrates backward perturbations, whereas the right column presents forward perturbations. The perturbation onset occurs at *t* = 0 s. Blue dashed boxes indicate the theta frequency and temporal window of interest.

After applying GED to the (artifact corrected) EEG of each participant, we selected the GED component with the greatest eigenvalue, clear ERP average, and a midfrontal scalp topography. This resulted in midline scalp topographies for all participants (Fig. 4A; see for example Solis-Escalante et al. 2019).

Time–frequency analysis of the selected GED components averaged over all leaning conditions and participants revealed overall strong theta power increase for both perturbation directions and feet-in-place or stepping responses over the theta and alpha frequencies post perturbation onset (Fig. 4B). Theta dynamics were stronger and longer lasting for stepping responses compared to feet-in-place responses. The theta frequency over 0.1–0.3 s is indicated with a blue dashed box. Baseline theta power was similar for all leaning conditions (*F*(2) = 0.61, *P* = 0.55), indicating that theta power was not affected by the leaning prior to perturbation onset.

### GLME model fit of cortical dynamics

In addition to theta dynamics, similar analyses were conducted on the alpha and beta range on the forward perturbation model (see Supplementary Material).

#### Forward perturbation direction theta dynamics model

**Fig. 5:**
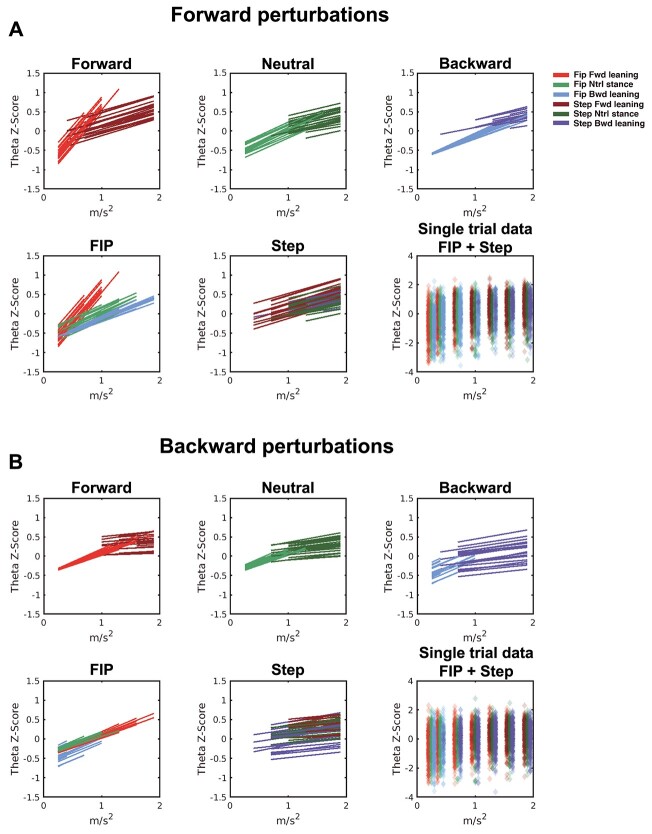
Theta power model fit. A) Forward perturbation model fit of single-trial theta dynamics. The top row shows theta dynamics of each leaning condition with fip (lighter color) or step response (darker color). In the bottom row, all leaning condition fip responses are presented left and step responses in the middle. The bottom right panel shows single-trial normalized theta power for each participant per leaning condition. A small offset on the *x*-axis was added to represent the 3 leaning conditions in the same platform acceleration bin. B) Backward perturbation model fit of single-trial theta dynamics. Data presented as forward leaning (red), neutral stance (green), and backward leaning (blue).

The forward perturbation direction GLME model was overall a highly significant fit to the data (*R*^2^ = 0.147, *F*(3703) = 29.70, *P* = 1.27e^−60^) (Fig. 5). There was a main effect of platform acceleration indicating greater theta power associated with higher platform accelerations (${\beta}_1$ = 0.622, CI: [0.49 0.76], *P* = 9.11e^−19^). An effect of “Forward leaning condition” (${\beta}_3$ = −0.37, CI: [−0.63–0.11], *P* = 4.76e^−3^) indicated that theta power was lower for backward leaning (most stable condition) than for forward leaning (least stable condition) at the same platform accelerations. There was an interaction effect of “Acceleration × Forward leaning” (${\beta}_4$ = 1.26 CI: [0.78 1.75], *P* = 2.91e^−7^), indicating greater increase of theta power with platform accelerations when leaning forward versus leaning backward. Furthermore, there was an “Acceleration × Forward leaning × Stepping” interaction (${\beta}_7$ = −1.13, CI: [−1.79–0.46], *P* = 8.86e^−4^), indicating that the different slopes in theta power observed due to the interaction effect of “Forward leaning condition × Acceleration” were only observed for the feet-in-place responses and not for the stepping responses.

#### Backward perturbation direction theta dynamics model

The backward perturbation direction GLME model (*R*^2^ = 0.084, *F*(3697) = 11,58, *P* = 1.46e^−21^) showed a main effect of “Acceleration” (${\beta}_1$ = 0.56, CI: [0.40 0.72], *P* = 3.52e^−12^) and an interaction effect for “Acceleration × Stepping” (${\beta}_5$ = −0.40, CI: [−0.79–0.02], *P* = 0.04). This interaction indicates that the slope of theta power is different for the stepping responses compared to feet-in-place responses (i.e. steeper when maintaining feet-in-place; Fig. 5). Interestingly, in the backward perturbation direction, we did not find a main or interaction effect for the leaning conditions.

## Discussion

Our goal was to demonstrate the participation of midfrontal theta dynamics in the behavioral monitoring of standing balance. We used whole-body balance perturbations of varying accelerations to probe the postural control system while manipulating the initial state of postural stability with a leaning task. This experimental paradigm allowed us to investigate the theta dynamics during altered states of postural stability. We found that different leaning conditions changed the relation between platform acceleration and midfrontal theta power. In particular, we found that the initial postural state, as imposed by the leaning instructions, led to different theta power modulations at identical platform accelerations. This is supporting evidence for the role of the midfrontal theta dynamics in balance monitoring and highlights that the role of midfrontal theta is nuanced and dependent on contextual postural factors.

### Evidence of a monitoring role of midfrontal theta dynamics

We, and others, have speculated that midfrontal theta during balance monitoring reflects the activation of a cortical system that compares incoming postural information to an internal model of postural stability, and predicts the need for a corrective step. Consistent with the suggested monitoring role of midfrontal theta dynamics, the midfrontal theta power increases prior to loss of balance in a balance beam walking paradigm (Sipp et al. 2013), near the instant of minimum MoS (Slobounov et al. 2009), and during continuous challenges to postural stability (Hülsdünker et al. 2015). In response to balance perturbations, theta power dynamics scale with perturbation intensity (Varghese et al. 2014, 2019; Payne and Ting 2020) and with different reactive responses (i.e. feet-in-place versus stepping, Solis-Escalante et al. 2020), suggesting that theta dynamics scale with task difficulty. Our current results demonstrate that the relation between theta power with acceleration and the ensuing postural response can be altered by the leaning condition (i.e. initial postural state). In the forward perturbation direction, the relation between theta power with acceleration and the postural response significantly changed when leaning forward (in contrast to leaning backward, see bottom left panel in Fig. 5). In particular, when being perturbed in the forward direction, theta power increases more rapidly over acceleration when leaning forward and maintaining feet-in-place compared to leaning backward. Importantly, we do not observe changes in theta dynamics during baseline periods of the different leaning conditions, indicating that theta dynamics are not altered during the leaning phase prior to perturbation resulting in the observed differences. In addition, analysis of alpha and beta frequency bands (Supplementary Material) did not show similar interaction effects, suggesting that only theta dynamics scale with behavioral balance monitoring parameters.

We propose that the leaning posture alters the internal representation of postural stability in such a way that balance perturbations of a given intensity and direction are perceived as different postural threats and may elicit distinct postural responses. The theta dynamics observed in the range of accelerations that elicit feet-in-place responses reflect the rapid increase in postural threat associated with perturbations toward the leaning direction. Following the perturbation onset, incoming sensory information (representing the perturbation characteristics) may be quickly integrated within the central nervous system and compared against the estimated sensory information (given by the internal model of postural stability; see Scott 2004; Forbes et al. 2018; Rasman et al. 2018); any deviation from the internal estimate would require adaptive postural responses, and the cortical involvement in this computation manifests as midfrontal theta (Solis-Escalante et al. 2020). An important finding of the present study is that these theta dynamics depend on the initial postural situation in addition to the magnitude of the induced perturbation when feet-in-place responses are still feasible. An effect of initial leaning posture on midfrontal theta dynamics is not observed at higher perturbation intensities, where stepping responses are consistently elicited, and therefore, the midfrontal theta may have reached a threshold value indicating that a feet-in-place strategy is insufficient to maintain balance.

Interestingly, the balance-related cortical theta dynamics show strong similarities to cortical theta dynamics during other action monitoring paradigms. Many paradigms investigating action monitoring during dynamic tracking tasks show modulations of the midfrontal theta rhythm with similar latencies and scalp distributions to the results presented in our study (Cohen and Donner 2013; Cavanagh and Frank 2014). An increase of midfrontal theta power coincides with behavioral errors, and the magnitude of these errors (as well as the ensuing corrective behavior) corresponds with the magnitude of theta power (Pereira et al. 2017). In our study, the theta dynamics preceded the stepping responses and seemed to represent active monitoring of balance, which is most relevant when perturbation acceleration and initial leaning condition lead to feet-in-place responses.

### Balance monitoring and perturbation direction

Our analyses of midfrontal theta dynamics revealed an effect of the initial leaning condition in the forward perturbation direction, but not in the backward perturbation direction. Albeit our goal was not to compare between the 2 postural perturbation directions, our results suggest that the initial postural state given by the leaning conditions prior to perturbation onset is less relevant for the backward perturbation direction. This may be explained by different anatomical and biomechanical constraints in the backward direction. The musculoskeletal system is primarily built to propel the human body in the forward direction. This can be observed in the greater distance of the ankle joint to the toes versus its distance to the heels, which creates a larger lever arm and allows generating greater ankle plantar flexion than dorsiflexion torques, and the greater range of motion of the hip joint in flexion than extension. These differences offer clear advantages for balance recovery in the forward direction. Ankle strategies help to maintain balance when CoM displacement is small; however, when the CoM displacement is larger, hip strategies help counteracting the CoM acceleration to prevent humans from falling forward. As these strategies are not applicable to the same extent for the backward perturbation direction, postural perturbations of equal magnitude lead to a higher likelihood of stepping responses in the backward than in the forward perturbation direction.

To prevent falls in the backward direction, it seems most effective to initiate stepping responses not only at lower platform intensities, but also sooner after perturbation. Indeed, we found that stepping latencies occurred faster in the backward direction (376 ms) compared to the forward direction (440 ms). Interestingly, in our previous study (Solis-Escalante et al. 2020), we found no difference in forward and backward perturbations N1 latency, which suggests that monitoring postural stability occurs with a similar interval for forward and backward perturbation directions, but later response onset in the forward perturbation direction leads to more time available to determine the necessity of a stepping response (c.f. Manista and Ahmed 2012). Therefore, we speculate that in the backward direction, the shorter time until step onset may limit the role of midfrontal theta modulations in selecting the behavioral response, which appears in line with the overall lower explained variance of the backward perturbation direction GLME model (8.4% vs. 14.7% for the forward perturbation direction model).

Whether balance is monitored differently for perturbation directions has remained elusive. Despite previous studies reporting that the perturbation direction does not modulate the perturbation-evoked responses (Dietz et al. 1985; Goel et al. 2018; Payne et al. 2019), recent studies suggest a cortical representation of direction-specific postural stability (Solis-Escalante et al. 2020). Our results indicate that for the backward perturbation direction, the initial leaning posture does not change the relation between perturbation acceleration and theta power (see Fig. 5). We expected to observe a strong modulation of the midfrontal theta power during feet-in-place responses related to the initial leaning posture and indicating balance monitoring. Such modulation of the midfrontal theta power with initial leaning posture was indeed observed in the forward perturbation direction (see top right panel Fig. 5), yet the larger proportion of stepping responses in the backward perturbation direction (see Section 3.1) may have obscured a similar effect of leaning posture. On the other hand, the slopes of the backward perturbation direction in feet-in-place data in the top-left panel of Fig. 5 seem to follow a parallel trend and do not seem to show a threshold of theta power indicating the necessity of a step.

### Clinical implications

Postural balance control is an ongoing cognitive-motor process that all humans engage in every day of their lives. Impairments in postural control can be devastating to quality of life. In fact, instability (leading to high fall risk) is a major problem in people with neurological disorders, as well as in the rapidly growing older population. Despite the severity of this problem and the pivotal role of the brain in controlling the musculoskeletal system, we still have a remarkably poor understanding of the cortical mechanisms involved in postural control. Our present suggestion of theta activity representing action monitoring of postural balance according to an internal model of stability informs future studies where, for instance, we may want to seek further evidence for this hypothesis in patients with known deficits in internal modeling (e.g. cerebellar pathology). In addition, it may be of interest to investigate whether aberrant internal modeling may underlie (or contribute to) postural instability and falls in various neurological conditions (e.g. stroke or Parkinson’s disease). For such studies, it is recommended to limit the experimental duration and perform a small subset of perturbations, while still allowing for a sufficient number of repetitions at each experimental condition. As we showed most cortical modulations related to balance monitoring at the lower end of the spectrum of perturbation intensities, perturbations may largely constitute those eliciting feet-in-place responses. In addition, it may be helpful to use 2 contrasting leaning conditions, instead of the 3 conditions as used in the present experiment. Importantly, for such clinical experiments, a sophisticated experimental setup such as the Radboud Falls Simulator is not a prerequisite since there are affordable treadmill-based alternatives for delivering standardized anterior–posterior perturbations. The current study provides valuable insights on the specific experimental conditions that may be used to target cortical balance monitoring processes.

## Future studies

We recommend that future studies investigate a wider range of low acceleration balance perturbations in the backward perturbation direction with different leaning postures, to investigate the effect on the modulation of midfrontal theta power in the backward perturbation direction. The current study illustrates that the cortical role in balance monitoring does not solely represent the characteristics of the perturbation itself but also accounts for contextual information (i.e. postural threat). This provides supporting evidence for future studies to investigate to what extent different sensory modalities contribute information to the internal model of stability.

## Limitations

Changes in sensory information due to perturbation intensity and through the leaning conditions are hard to disentangle. Experimental manipulations of sensory inflow (e.g. vibration to Achilles tendon) would not allow for perfect dissociation with perturbation magnitude, due to reweighting mechanisms of the highly redundant sensory inputs that would remain unaffected by such manipulations (e.g. exteroceptive information from the foot soles). Therefore, one can only speculate how sensory information is altered by the induced leaning conditions and whether this might have interacted with observed balance monitoring theta dynamics. Noteworthy, our experimental manipulations maintained the intensity of external balance perturbations while allowing for natural (ecologically valid) sensory reweighting of the postural control system. An important finding of the present study is that these theta dynamics depend on the initial postural situation in addition to the magnitude of the induced perturbation when feet-in-place responses are still feasible.

In addition, the time window allows us to interpret theta dynamics in response to the imposed postural threats following the 100–300 ms post perturbations. Yet, postural balance control is a dynamic process with cortical dynamics changing over time. Although, the selected time window in this study may help explain the role of theta dynamics during the monitoring of postural balance, it should be mentioned that dynamics outside of this window may also contribute to the monitoring of postural balance.

While, at first glance, the GLME model fit seems to suggest that feet-in-place responses elicit greater theta dynamics compared to step responses for the highest accelerations resulting in feet-in-place responses, there are several factors that preclude making this inference. Primarily, we would like to point out there was an unequal amount of observations in either feet-in-place and step response per acceleration bin (i.e. gradually decreasing numbers of feet-in-place trials with increasing perturbation intensities, and vice versa for stepping trials). As the GLME model fit yields more uncertainty for the estimates at the extreme ends of the spectrum with low numbers of trials, it may have overestimated these feet-in-place response theta dynamics.

Another point of attention may be the differences in the stepping ratios for forward and backward perturbation directions, as we found significantly more step responses in the backward perturbation direction. Although the amount of responses seems more balanced in the backward direction compared to the forward direction, we expected that the leaning effect on cortical dynamics of balance monitoring to be more pronounced during feet-in-place trials. As stepping thresholds are lower in the backward direction, the current experimental setup may not have been adequate to elicit these monitoring dynamics in the backward direction. Therefore, backward perturbations at low intensities and small increments may illustrate similar balance monitoring dynamics.

## Authors’ contribution

Mitchel Stokkermans : Designed research, analyzed data, and wrote paper. Teodoro Solis-Escalante, Michael X Cohen, and Vivian Weerdesteyn: Supervised Mitchel Stokkermans throughout the study and provided feedback.

## Supplementary Material

Supplementary_material_bhac283

## Data and code availability statement

The data and code will be made available through a public repository.
