# Supplementary material for: Midfrontal theta dynamics index the monitoring of postural stability
Source: Cereb Cortex. 2022 Sep 6;33(7):3454–66. doi: 10.1093/cercor/bhac283 (PMC10068289; doi:10.1093/cercor/bhac283)
Supplement: Supplementary_material_bhac283 [file supplementary_material_bhac283.docx]

Supplementary material

Manuscript: Midfrontal theta dynamics index monitoring postural stability

Statistical analysis of the cortical alpha (9 – 12 Hz) and beta (15 – 25 Hz) frequency range was conducted similarly to the theta power GLME analysis for the forward perturbation direction. These results indicate that only theta dynamics facilitate the monitoring of human balance control.

***Alpha dynamics***

In the additional analysis of the alpha (9 – 12 Hz) frequency band range (R^2^ = 0.07, F(3703) = 16,24, p=1.28e^-31^, figure S1), we do observe main and interaction effects of *Acceleration x Stepping* (β_5_ = -0.48, CI: [-0.88 -0.08], p = 0.02), indicating that over all leaning conditions the slope of theta under feet-in-place responses is steeper than for step responses (observed in the top row of figure 2). In addition, we observe an interaction effect of *Acceleration x Leaning forward* (β_4_ =0.58, CI: [0.10 1.1], p = 0.02), indicating that when leaning forward, alpha power increases faster over accelerations compared to leaning backward. However, there is no three-way interaction observed in slope between feet-in-place and step responses when leaning forward. The lack of an effect between feet-in-place and stepping response when leaning forward suggests that alpha only signals for an increase in postural threat explained by acceleration and leaning but not with the ensuing behavioral response as theta dynamics do.


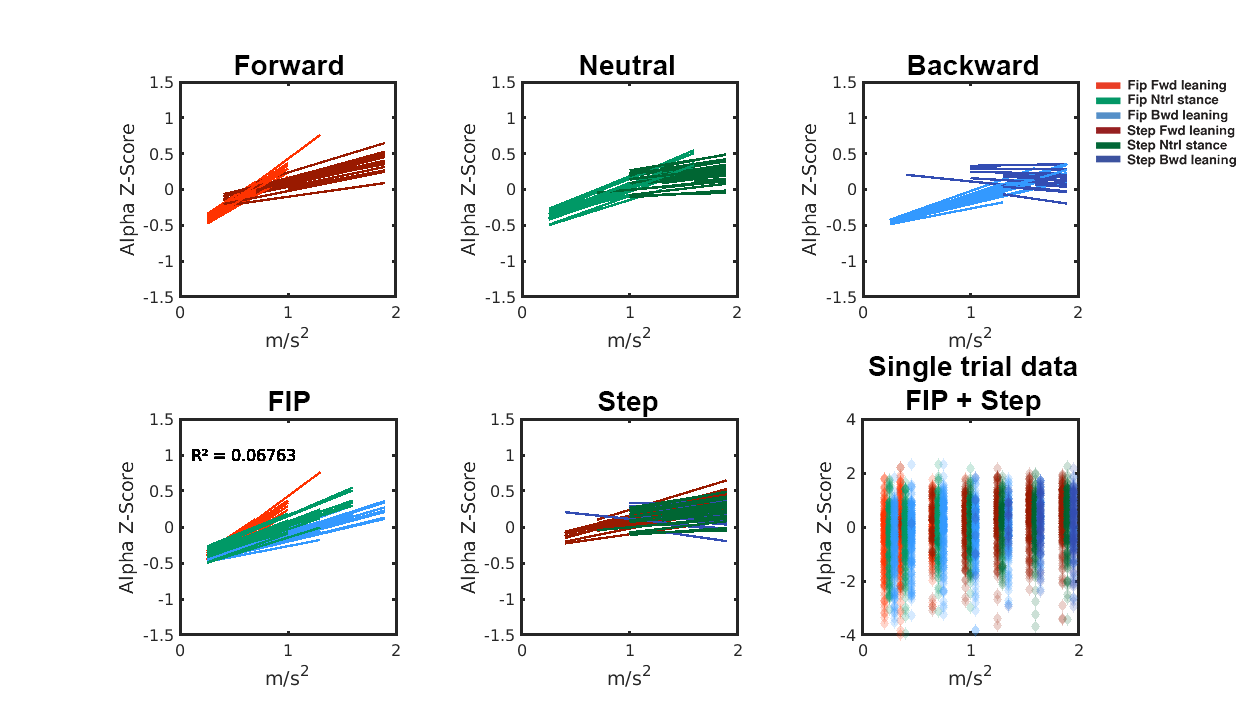


Figure S1: Alpha dynamics GLME model fit. The top row illustrates the different leaning conditions forward leaning (left), neutral stance (middle), backward leaning (right) with both feet-in-place (fip) (lighter color) and step response (darker color). The bottom row contains the fip response (bottom left) and the step response (middle) of the three leaning conditions. In the bottom right panel, the single trial data is presented with an offset on the x-axis to illustrate all three leaning conditions per acceleration bin.

***Beta dynamics***

In the beta (15 – 25Hz) frequency range (R^2^ = 0.03^­^, F(3703)=9.9, p=6.6e^-18^_,_ figure S2) we observe an effect of *Acceleration* (β_1_ = 0.21, CI: [0.075 0.35], p = 0.002). In addition, we observe an interaction effect of *Stepping x Leaning* for both forward (β_6_ = -0.96, CI: [-1.79 -0.13], p = 0.02) and at neutral stance (β_6_ = -95, CI: [-1.85 -0.05], p = 0.04). This indicates that the slope of beta dynamics is different when leaning backward and stepping compared to the other leaning postures (observed in the bottom middle graph of figure s2). In addition, a three-way interaction is observed (β_7_ =0.66, CI: [0.077 1.24], p = 0.03) which indicates that the slope of beta dynamics is different when leaning backward and stepping compared to feet in place. This is observed as the negative slope for backward leaning and stepping, compared to feet in place in the top right graph of figure s2.


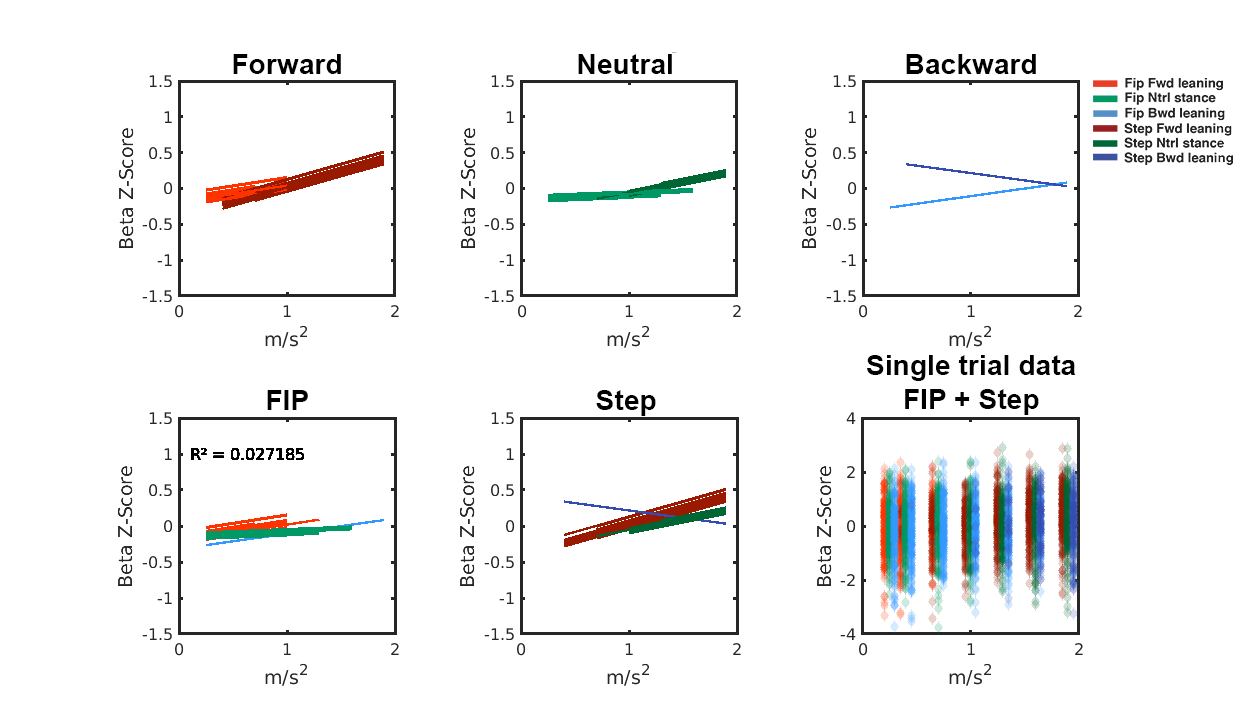


Figure S2: Beta dynamics GLME model fit. The top row illustrates the different leaning conditions forward leaning (left), neutral stance (middle), backward leaning (right) with both feet-in-place (fip) (lighter color) and step response (darker color). The bottom row contains the fip response (bottom left) and the step response (middle) of the three leaning conditions. In the bottom right panel, the single trial data is presented with an offset on the x-axis to illustrate all three leaning conditions per acceleration bin.

Although we observe a three-way interaction in the beta range, these observed interactions concern differences in beta dynamics for stepping between forward and backward leaning. Importantly, we do not consider this as monitoring of balance as we expect markers for monitoring to scale within the feet-in-place responses (when any response outcome is still possible), rather than the step responses at observed in the beta dynamics. Within the feet-in-place response there is a stability range to monitor postural balance before balance is lost, whereas in stepping trials the possibility for a feet-in-place response is exceeded and always results in the loss of stability and a step response.
